# Immunization with Heat Shock Protein A and γ-Glutamyl Transpeptidase Induces Reduction on the *Helicobacter pylori* Colonization in Mice

**DOI:** 10.1371/journal.pone.0130391

**Published:** 2015-06-23

**Authors:** Xiaoli Zhang, Jinyong Zhang, Feng Yang, Weiru Wu, Heqiang Sun, Qinghua Xie, Weike Si, Quanming Zou, Zhong Yang

**Affiliations:** 1 Department of Clinical Hematology, Southwest Hospital, Third Military Medical University, Chongqing 400038, PR China; 2 National Engineering Research Center of Immunological Products, Department of Microbiology and Biochemical Pharmacy, College of Pharmacy, Third Military Medical University, Chongqing, 400038, PR China; Instituto Butantan, BRAZIL

## Abstract

The human gastric pathogen *Helicobacter pylori* (*H*. *pylori*) is a successful colonizer of the stomach. *H*. *pylori* infection strongly correlates with the development and progression of chronic gastritis, peptic ulcer disease, and gastric malignances. Vaccination is a promising strategy for preventing *H*. *pylori* infection. In this study, we evaluated the candidate antigens heat shock protein A (HspA) and *H*. *pylori* γ-glutamyl transpeptidase (GGT) for their effectiveness in development of subunit vaccines against *H*. *pylori* infection. rHspA, rGGT, and rHspA-GGT, a fusion protein based on HspA and GGT, were constructed and separately expressed in *Escherichia coli* and purified. Mice were then immunized intranasally with these proteins, with or without adjuvant. Immunized mice exhibited reduced bacterial colonization in stomach. The highest reduction in bacterial colonization was seen in mice immunized with the fusion protein rHspA-GGT when paired with the mucosal adjuvant LTB. Protection against *H*. *pylori* colonization was mediated by a strong systemic and localized humoral immune response, as well as a balanced Th1/Th2 cytokine response. In addition, immunofluorescence microscopy confirmed that rHspA-GGT specific rabbit antibodies were able to directly bind *H*. *pylori in vitro*. These results suggest antibodies are essential to the protective immunity associated with rHspA-GGT immunization. In summary, our results suggest HspA and GGT are promising vaccine candidates for protection against *H*. *pylori* infection.

## Introduction

*Helicobacter pylori* (*H*. *pylori*), a spiral-shaped gram negative bacterium that colonizes the stomach of more than 50% of the world’s population, is the cause of chronic gastritis and peptic ulcers and is a risk factor for gastric cancer [1]. This pathogen could not be effectively cleared or prevented from re-infection by host immune system after successful antimicrobial treatment, thus it usually causes chronic infection, with colonization persisting for the lifetime of the host [2].

When *H*. *pylori* have been detected in patients with gastric disease, the normal procedure is to eradicate the bacteria in order to cure the disease. The standard treatment is a proton pump inhibitor like Omeprazole, and the antibiotics clarithromycin and amoxicillin for one week [3]. However, due to drawbacks like antibiotic resistance, adverse reactions to treatment, re-infection and poor patient compliance, antibiotic therapy does not always work well [4]. For these reasons, in order to prevent infection or treat and already established infection, vaccination is considered a promising and reliable alternative approach for the clinical management of *H*. *pylori* infections.

Since *H*. *pylori* was first identified in 1983 [5], researchers have sought after a vaccine to protect against infection by this bacterium. Many types of vaccines have been developed over the past two decades, including whole cell vaccines, subunit vaccines, live vector vaccines, DNA vaccines, and epitope vaccines [6,7]. While many of these experimental vaccines have been tested in animal models, only a few have reached clinical trials, and none have obtained market authorization [8]. Among the many vaccines assessed in animal models and clinical trials, subunit vaccines seem to be the most promising category. As such, subunit vaccines are still under extensive investigation. Many candidate antigens have been identified in *H*. *pylori*, such as HpaA, UreB, NapA, Lpp20, CagA and VacA [9–13]. Already tested vaccines composed of these antigens, however, do not afford complete protection [6]. Thus, efforts to screen and identify more immunogenic and effective antigens are urgently required.

HspA has long been considered as a candidate antigen for vaccine development. Ferrero RL *et al* reported in 1995 that HspA confers protective immunity against *H*. *pylori* infection [14]. As an alternative, *H*. *pylori* γ-glutamyl transpeptidase (GGT) is a new, highly conserved virulence factor that was identified recently. Although the immunogenicity of GGT has not yet been reported, its homologue in *Haematopinus suis* provides protective immunity against infection when immunized in combination with UreB [15]. Since both candidate antigens, HspA and GGT, provide partial protection against *H*. *pylori* infection, we sought to determine whether combining both antigens would produce a more effective vaccine.

In this study, we systematically evaluate the effectiveness of HspA and GGT as candidate antigens for *H*. *pylori* vaccine development. Both antigens were separately expressed in *E*. *coli*, or expressed as a fusion protein. Then, HspA, GGT, or the fusion protein was immunized intranasally with different adjuvants, and the ability to induce mucosal and system immunity, as well as any effect on protective immunity, was evaluated in a mouse model of *H*. *pylori* infection.

## Materials and Methods

### Ethics statement

All animal care and use protocols were performed in accordance with the Regulations for the Administration of Affairs Concerning Experimental Animals approved by the State Council of People's Republic of China. All animal experiments were approved by the Animal Ethical and Experimental Committee of the Third Military Medical University (Chongqing, Permit No. 2011-04) in accordance with their rules and regulations.

### Construction, expression and purification of recombinant proteins

**Fig 1:**
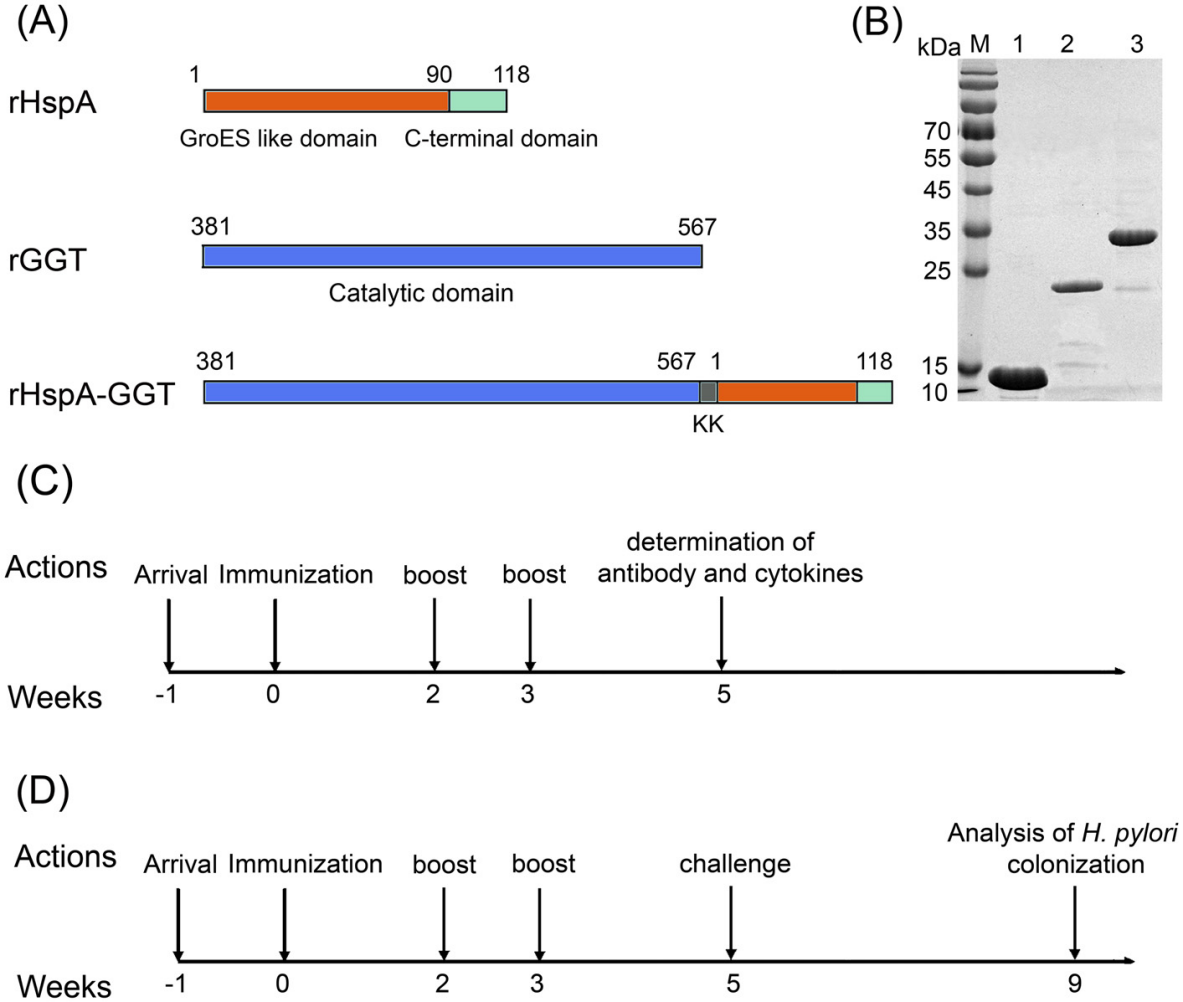
Candidate antigens and immunization schedule used in this study. (A) Three His-tagged recombinant proteins were designed to be used as candidate antigens: full length HspA (rHspA), the catalytic domain of GGT (rGGT), and a fusion protein composed of rHspA and rGGT linked by two lysine residues (rHspA-GGT). (B) Recombinant proteins were purified by nickel ion affinity chromatography and gel-filtration chromatography. Recombinant proteins were then analyzed by SDS-PAGE. (C) The schedule for vaccine immunization, determination of cytokines secretion, and antigen-specific antibodies. (D) The schedule for vaccine immunization and analysis of *H*. *pylori* colonization.

As shown in Fig 1A, three recombinant proteins: rHspA (full length), rGGT (amino acids 381 to 567 that correspond to the catalytic domain of GGT), and a fusion protein rHspA-GGT (full length HspA fused to the catalytic domain of GGT by a “KK” linker) were constructed in this study. The coding sequences of rHspA and rGGT were directly amplified from the genome of *H*. *pylori* strain 26695, by PCR, then cloned into an expression vector derived from the pET30a(+) plasmid (Novagen), and placed between *NdeI* and *XhoI* restriction sites. The plasmid pET30a-rHspA-GGT was synthesized by Sangon by overlapping PCR (China), primers used in this study were listed in S1 Table. All recombinant plasmids were transformed into *E*. *col*i BL21 (DE3) pLysS cells (Invitrogen), and protein expression was induced with 1 mM IPTG. Cells were harvested by centrifugation and bacterial pellets were resuspended in lysis buffer (20 mM phosphate buffer pH 8.0, 300 mM NaCl, and 10 mM imidazole). Resuspended cells were disrupted by ultrasonication. Any insoluble cellular material was removed by centrifugation. Then, the supernatant was loaded onto a nickel nitrilotriacetic acid agarose (Ni–NTA) column (Novagen) that had been previously equilibrated with lysis buffer. The column was washed with wash buffer (20 mM phosphate buffer pH 8.0, 300 mM NaCl, and 20 mM imidazole). His-tagged proteins were then eluted using elution buffer (20 mM phosphate buffer pH 8.0, 300 mM NaCl, and 250 mM imidazole) followed by gel-filtration on a Superdex 200 HiLoad 16/60 column (GE Healthcare) previously equilibrated with buffer containing 20 mM PBS pH 7.5 and 150 mM NaCl. The peaks corresponding to the target proteins were collected for each recombinant protein and lipopolysaccharide (LPS) contamination were further removed by ion-exchange chromatography. The recombinant proteins were analyzed by SDS-PAGE, and the concentrations of the purified proteins were determined by BCA assay.

### Immunization and infection

Six to eight-week-old SPF female BALB/c mice were purchased from the Experimental Animal Center of Third Military Medical University. Immunization and infection experiments were performed as shown in Fig 1C and 1D. Briefly, to quantify antibody and cytokine production, mice were randomly divided into 6 groups (n = 5) and intranasally immunized three times on days 0, 14, and 21. Vaccine formulations consisting of different antigens and adjuvants (LTB and CPG) were dissolved in PBS in a total volume of 20μl (Table 1). Mice were sacrificed on day 35, and sera were collected to analyze specific IgG antibody responses. Gastric and small intestine tissue samples were collected as described previously [16] to analyze the local sIgA response. Spleen cells were also collected to analyze cytokine production. In order to evaluate each vaccine formulation’s protective efficacy against *H*. *pylori* infection, mice in each group (n = 10) were orally challenged four times with 10^9^ CFU of the BALB/c mouse-adapted *H*. *pylori* strain B6 two weeks after the last boost. Four weeks after this challenge, mice were sacrificed and the stomachs were separated to determine the amount of bacterial colonization.

**Table 1:** Vaccine formulations in each group.

Group	rHspA(μg)	rGGT(μg)	rHspA-GGT(μg)	LTB(μg)	CpG(μg)	Total volume(μl)
PBS	0	0	0	0	0	20
CpG	0	0	0	0	10	20
LTB	0	0	0	10	0	20
rHspA+LTB	30	0	0	10	0	20
rGGT+LTB	0	30	0	10	0	20
rHspA-GGT+LTB	0	0	30	10	0	20
rHspA-GGT+CpG	0	0	30	0	10	20
rHspA-GGT	0	0	30	0	0	20

### Antibody production assay

Antibody titer of the collected sera was determined by ELISA as described previously [17]. 96 well flat bottom plates were coated with *H*. *pylori* 26695 lysate (2 μg/ well) and purified rHspA-GGT (1 μg/well). After blocking with 5% bovine serum albumin (BSA) buffered with PBS, 100 μL of 1:500 diluted serum was added to each well as the primary antibodies. The appropriate HRP-labeled anti-mouse IgG and its subtypes IgG2a and IgG1 were used as secondary antibodies. Absorbance was read at 450 nm (OD_450_). All samples were tested in triplicate. Gastric and small intestine samples containing sIgA antibodies were collected, and the level of specific sIgA was subsequently measured by ELISA, as described above.

### Analysis of *H*. *pylori* colonization

Immunized and control mice were sacrificed in order to quantify *H*. *pylori* stomach colonization by real-time PCR using the TaqMan method. Stomachs were homogenized in PBS and DNA was extracted using the QIAamp DNA Mini Kit (Qiagen Inc., Valencia, CA, USA). We amplified *H*. *pylori* 16S rDNA as described previously [24]. Briefly, the primers used were F: 5’-TTTGTTAGAGAAGATAATGACGGTATCTAAC-3’ and R: 5’-CATAGGATTTCACACCTGACTGACTATC-3’, and the TaqMan probe was P: 5’-FAM-CGTGCCAGCAGCCGCGGT-TAMRA-3’. Real-time PCR was carried out on a Bio-Rad iQ5 multicolor Real-time PCR Detection System using the absolute quantification option. After denaturation at 94°C for 3 min, samples were amplified at 94°C for 15 seconds and 65°C for 1 minute for 35 cycles.

### Lymphocyte cytokine response assay

Mice were sacrificed and spleens were harvested in order to quantify the lymphocyte cytokine response. Splenic cells were collected and resuspended at a concentration of 2×10^6^ cells/ml in RPMI 1640 medium supplemented with 10% fetal calf serum (FBS, HyClone Laboratories). The spleen cells were incubated at 37°C with 5 μg/ml of rHspA-GGT. After 3 days of incubation, samples were centrifuged and the supernatant were collected. IFN-γ, IL-4, IL-5, and IL-17A cytokine levels in the supernatants were quantified by specific ELISA kits (Dakewe) following the manufacturer’s instructions. The concentrations were calculated relative to supplied, calibrated cytokine standards, and expressed in pg/ml units.

### Generation of rHspA-GGT specific polyclonal antibody

rHspA-GGT specific polyclonal antibody (rHspA-GGT pcAb) were generated in New Zealand white rabbits based on a previously published method [18]. Briefly, rHspA-GGT mixed with an equal volume of Freund’s complete adjuvant (Sigma, America) was injected intradermally on the back and proximal limbs of the rabbit at days 0, 21, and 28. Blood was harvested two weeks after the last immunization and serum was obtained by centrifugation. The IgG antibody in the serum was purified by affinity chromatography with a protein A column (GE, America) and followed by desalting with PBS.

### Indirect immunofluorescence

Indirect immunofluorescence experiment was performed to confirm the direct binding of antigen specific serum with bacteria *in vitro* [18]. *H*. *pylori* 26695 was cultured, washed and resuspended in PBS, The bacteria was then fixed with 1% paraformaldehyde followed by centrifugation at 8000 ×g for 5 min, bacteria were washed three times with PBS, and resuspended in PBS containing 10% fetal bovine serum (FBS) and 1% NaN3. After washed with PBS for three times, rHspA-GGT pcAb and negative control pcAb was then added to tubes followed by incubation at 37°C for 1 h, rabbit polyclonal antibody to *H*. *pylori* urease B (Abcam, America), termed Ureb pcAb was used as a positive control for identification of *H*. *pylori*. Bacteria were washed three times with PBS, and bound antibodies were detected using FITC-labeled goat anti-rabbit IgG (Tianjin Sungene Biotech Co., Ltd.). Then bacteria were washed three times with PBS and resuspended in PBS containing 3% BSA, 1% NaN3 in darkness. The samples were analyzed by fluorescence microscopy (Ti-s, Nikon, Japan) and photos were taken immediately.

### Statistical analysis

All data were represented as mean ± standard deviation (S.D.). Means were compared using the two-tailed Students t-test, analysis were performed using GraphPad Prism 5.0 (GraphPad Software), and a P < 0.05 value was considered statistically significant.

## Results

### Recombinant protein expression and purification

The coding sequences of the three recombinant proteins (rHspA, rGGT, and rHspA-GGT) were inserted to the expression vector pET30a(+). Each insert was sequenced and confirmed to contain the correct sequence. Proteins were expressed in *E*. *coli* BL21 (DE3) pLysS, induced with 1 mM IPTG, and purified by affinity chromatograph and gel filtration, resulting in a high yield. As shown in Fig 1B, all three proteins were obtained with high purity (up to 90%) as determined by SDS-PAGE. The molecular weight of rHspA, rGGT and rHspA-GGT is 14.1, 21.4, and 34.2 kDa, respectively. The specific yield of rHspA, rGGT and rHspA-GGT in 1 L culture is about 20, 10 and 5 mg, respectively.

### Immunization with recombinant proteins correlates with reduced *H*. *pylori* colonization

**Fig 2:**
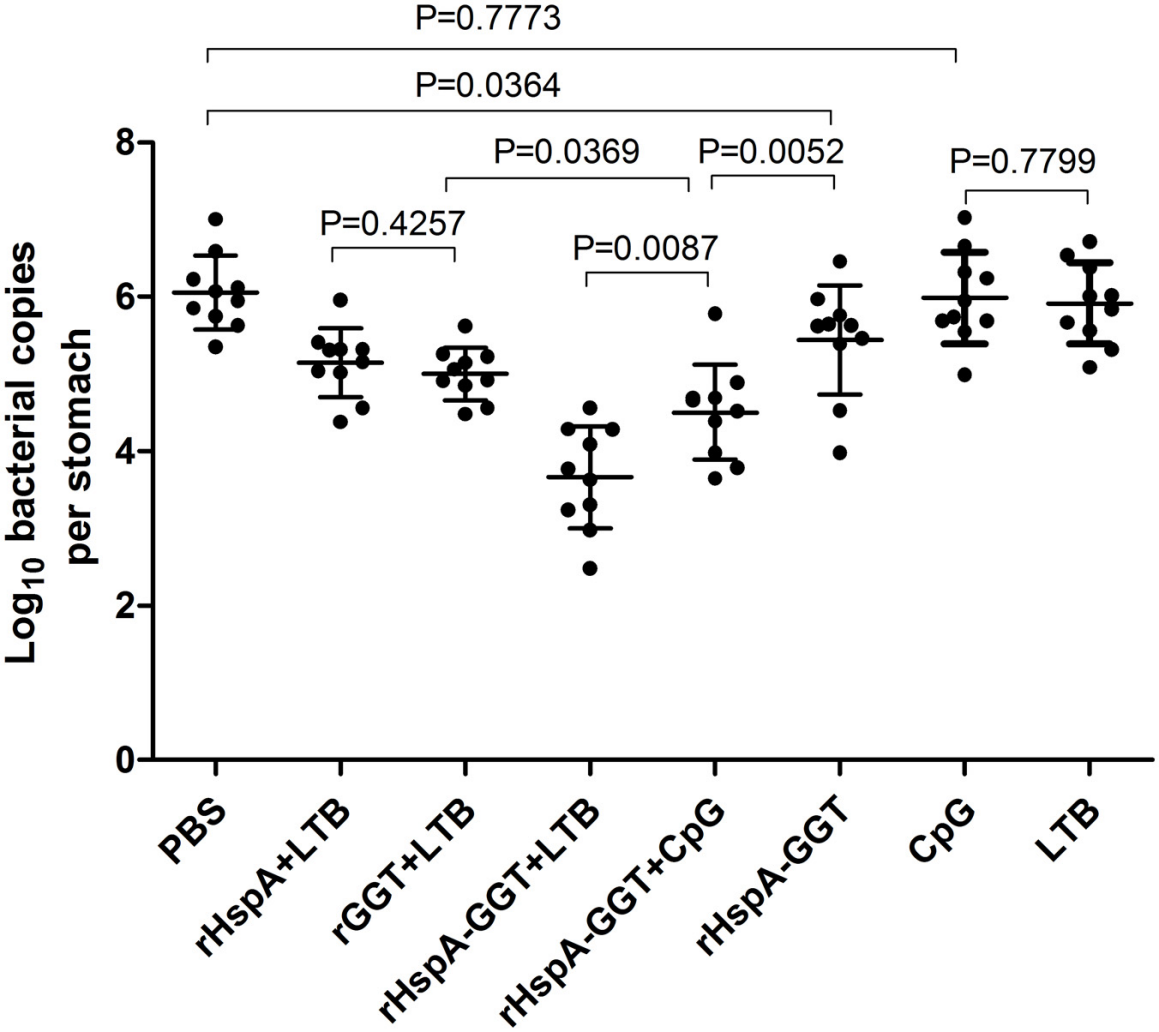
*H*. *pylori* colonization in mouse stomachs after immunization. BALB/c mice (n = 10) were immunized intranasally on days 0, 14 and 21 with 30 μg antigen, with or without 10μg adjuvant, as indicated in Table 1. The same volume of PBS was used as a negative control. Three weeks after the final vaccination boost, mice were orally challenged four times with *H*. *pylori* B6. The level of gastric *H*. *pylori* colonization was determined by real-time quantitative PCR four weeks post challenge for each mouse. Data are expressed as mean ± S.D. Significant differences between indicated groups are presented as P values.

In order to determine whether immunization with recombinant protein vaccines reduce bacterial load in the stomachs of *H*. *pylori-*infected mice, we quantified bacterial colonization in the stomach by real-time quantitative PCR four weeks after the mice were challenged. As shown in Fig 2, all immunizations that containing recombinant proteins induced a significant reduction in gastric bacterial load compared to PBS and adjuvant control group (P<0.05). Higher levels of protection were observed when an adjuvant, either LTB or CpG was used, indicating that adjuvants play an important role in inducing protective immunity in these experiments. The highest level of protection was seen in mice immunized with rHspA-GGT plus LTB. A 100 to 1000-fold reduction in bacterial load was observed compared with the PBS control group (P<0.0001). This amount of bacterial load was significantly lower than that of mice immunized with single antigens or mice immunized with the adjuvant CpG. These results indicate that the fusion protein elicited a stronger immune response in the mice, resulting in more bacterial clearance. The results also indicate LTB is superior to CpG in inducing protective immunity in these experiments (P<0.01). In contrast, after immunization with rHspA plus LTB or rGGT plus LTB, we observed an approximately 10-fold reduction in bacterial load, with no significant difference between these two conditions (P>0.05). These results validated the protective efficacy of HspA and GGT, and further confirmed that immunization with the fusion protein consisting of these two proteins is superior to either single antigen in eliciting a protective immune response.

### Antigen specific humoral immune response

**Fig 3:**
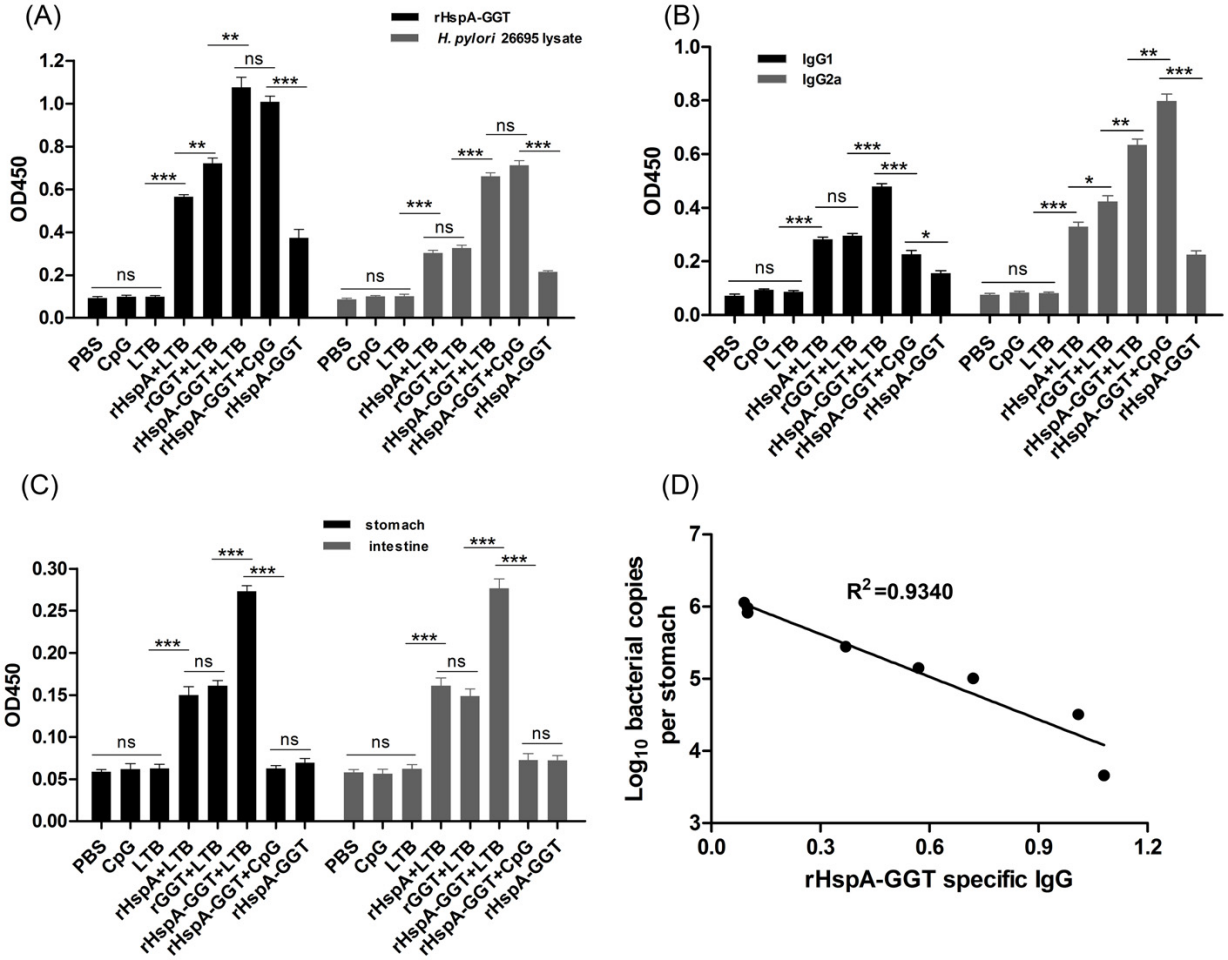
Antigen specific antibody responses elicited by immunization. Two weeks after immunization, mice in each group (n = 5) were bled and the sera were collected for analysis of the specific IgG antibody and its subtype. (A) Specific IgG antibody titers, for antibodies raised against rHspA-GGT and *H*. *pylori* 26695 lysate, were measured by ELISA. (B) The levels of specific IgG1 and IgG2a, against rHspA-GGT, were determined from serum samples tested by ELISA. (C) Immunized mice were sacrificed 4 weeks after challenge and the supernatants of homogenized stomachs and intestines were collected for detection of specific sIgA levels, against rHspA-GGT. Data are presented as mean ± S.D (n = 5). (D) Regression analysis of bacterial loads and the level of rHspA-GGT specific serum IgG. **P < 0.01 compared with indicated groups, ***P < 0.001 compared with indicated groups, ns: not significant.

Two weeks after the last boost immunization, sera from the mice were collected in order to determine the specific IgG antibody titer by ELISA. Fig 3A shows that both specific anti-rHspA-GGT and anti-*H*. *pylori* 26695 lysate IgG antibodies were significantly increased in animals immunized with antigens, with or without adjuvant, compared to PBS control group mice (P<0.01). The highest IgG levels were observed in mice immunized with the fusion protein rHspA-GGT. IgG levels in these mice were significantly higher than those of mice immunized with rHspA or rGGT alone, and no difference was observed in mice immunized with rHspA-GGT supplemented with either LTB or CpG adjuvant. In addition, mice immunized with the fusion protein lacking an adjuvant showed significantly lower serum IgG levels. Furthermore, a significant correlation (R^2^ = 0.9340) was observed between decreasing bacterial loads and increasing rHspA-GGT specific serum IgG levels (Fig 3D). These results indicate both rHspA and rGGT are strongly immunogenic and able to induce strong humoral immune response in mice. This may play an essential role in protection against *H*. *pylori* colonization. In addition, both LTB and CpG were able to enhance the humoral immune response to the fusion protein dramatically.

To determine the polarization of the immune response, the levels of rHspA-GGT specific IgG2a and IgG1 antibodies were also detected, to detect a possibly Th1 or Th2 biased immune response. As shown in Fig 3B, both IgG2a and IgG1 antibodies were significantly increased in the antigen-immunized groups compared with PBS control group. The IgG1/Ig2a ratio was 0.69 when immunized by rHspA-GGT without adjuvant, suggesting the presence of a balanced Th1/Th2 immune response was induced. In the three groups where LTB was used as an adjuvant, the calculated IgG1/Ig2a ratio range was from 0.69 to 0.85, indicating LTB has little impact in regulation of the immune response. In contrast, when CpG was used as an adjuvant, the IgG1/Ig2a ratio was 0.28. This result suggests CpG induces a Th1-biased immune response. Although different immune responses were observed for CpG and LTB, the total amount of IgG detected was similar for both adjuvants.

To evaluate the local humoral immune response induced by immunization, gastric and intestinal mucosal sIgA production was assayed for each vaccine formulation. As shown in Fig 3C, the three groups immunized with antigens plus LTB significantly elevated both gastric and intestinal mucosal sIgA compared to the other groups (P<0.0001). The other three groups exhibited no difference when compared to one another (P>0.05). Similarly, higher levels of sIgA were observed in mice immunized with the fusion protein than mice immunized with rHspA and rGGT (P<0.0001), suggesting the fusion protein is more efficient in inducing local humoral immune response.

Obviously, both CpG and LTB immunized alone exhibited no impact on antigen specific antibody secretion, neither IgG nor sIgA, although they may play a role in total antibody secretion.

### Vaccine induced lymphocyte responses

**Fig 4:**
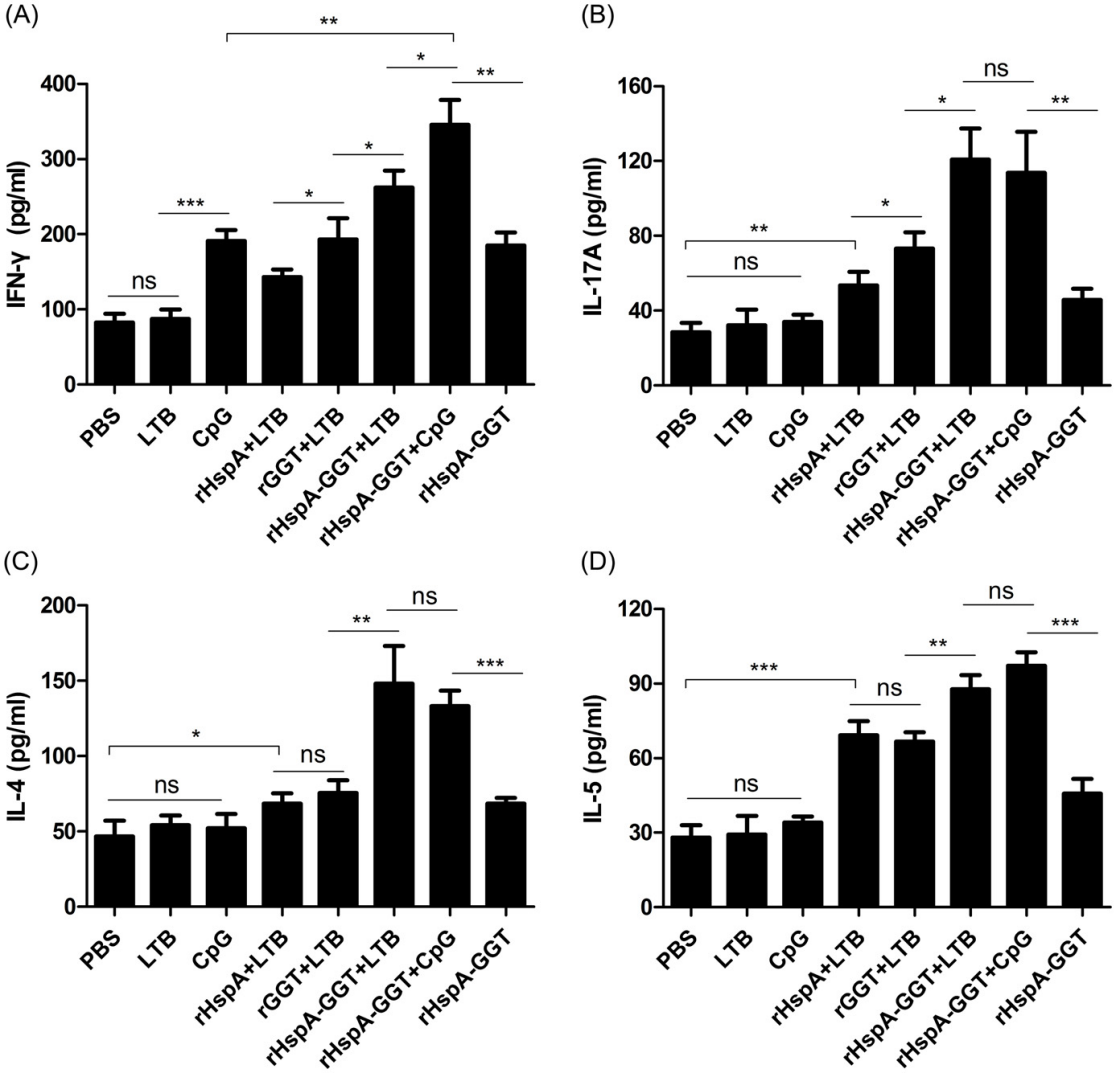
Immunization induced cytokine responses in murine spleen cells. Two weeks after the last booster, spleen cells of mice (n = 5) in each group were stimulated for 72 hours with antigen rHspA-GGT (5 μg/ml). The supernatants were harvested, and the cytokine levels of (A) IFN-γ, (B) IL-17A, (D) IL-4, and (D) IL-5 were determined by ELISA. Data are expressed as mean ± S.D. *P < 0.05 compared with all other groups or indicated groups, **P < 0.01 compared with indicated groups, ***P < 0.001 compared with indicated groups, ns: not significant.

The cytokine profiles in spleen cells from mice in each immunized group were determined two weeks after the last booster. Spleen cells from each group were stimulated with rHspA-GGT. Then, cytokine levels in the spleen cell supernatants were determined using ELISA. As shown in Fig 4, elevated levels of IFN-γ, IL-4, IL-5, and IL-17A were observed in all of the groups immunized with antigens when compared to the PBS control group, indicating a mixed Th1/Th2/Th17 response was induced upon vaccination. In the adjuvant control group, only mice immunized with CpG induced elevated levels of IFN-γ, whereas the other showed no difference as compared with mice immunized with PBS, indicating that antigens used in this study were efficient in eliciting cytokines secretion. Further, the levels of all tested cytokines were higher in mice immunized with the fusion protein when compared to the single proteins, and a higher level of cytokine response was observed in mice immunized with rHspA-GGT plus adjuvant compared to immunization without adjuvant. The highest amount of IFN-γ secretion was seen in mice immunized with rHspA-GGT supplemented with CpG (P<0.05 compared to all other immunization groups). These results indicate that CpG efficiently induces a Th1-biased response, consistent with the previous result wherein CpG induced more IgG2a antibodies than IgG1. The highest levels of the other three kinds of cytokines were observed in mice immunized with rHspA-GGT plus adjuvant, with no difference observed for the LTB and CpG supplemented groups. These results indicate these two adjuvants did not play a role in eliciting a Th2 and Th17 response. In addition, the level of IL-4 for each immunization group was similar to the level of IL-5, with both cytokines serving as representative cytokines for a Th2 response.

### Antigen specific IgG antibody was able to bind to *H*. *pylori* directly *in vitro*

**Fig 5:**
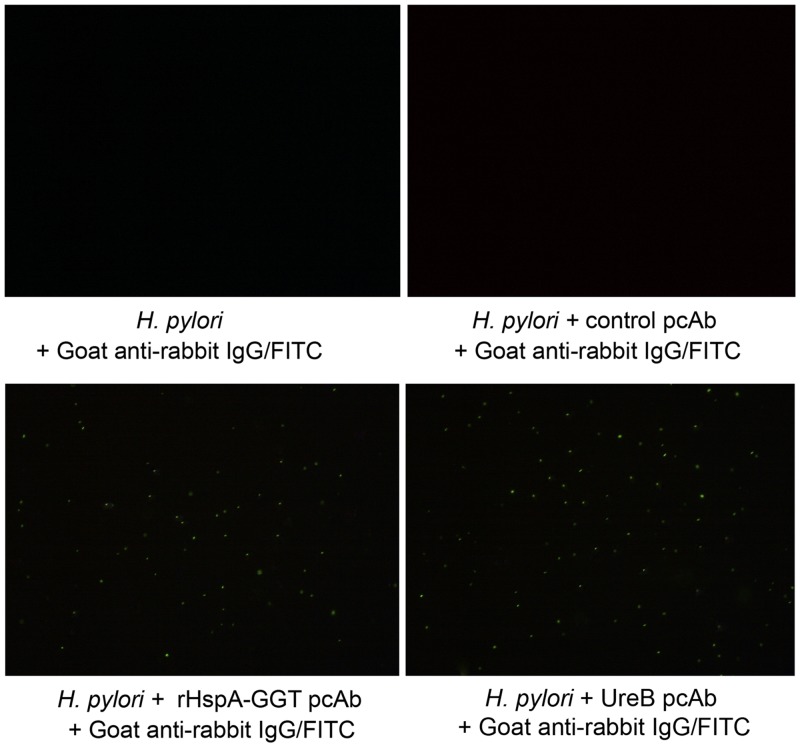
Indirect immunofluorescence confirmed that anti-serum collected from immunized mice directly binds *H*. *pylori in vitro*. No immunofluorescence was detected in the absence of antigen specific pcAb (A and B). Positive indirect immunofluorescence signals indicate binding of rHspA-GGT pcAb with *H*. *pylori* 26695 (C) and the existence of *H*. *pylori* (D). This study was performed twice, yielding similar results.

Since both HspA and GGT were reported to be surface exposed, and the latter is involved in bacterial colonization of the host stomach [19], we wondered if specific antibodies generated by these antigens could directly recognize *H*. *pylori in vitro*. In this study, rHspA-GGT pcAb was expressed and purified, and an indirect immunofluorescence assay was carried out to test for binding of rHspA-GGT pcAb to *H*. *pylori* 26695. Representative images are shown in Fig 5, positive indirect immunofluorescence was observed in the presence of Anti-urease B antibody, indicating the presence of the bacterial (Fig 5D). In contrast, no immunofluorescence was detected in the presence of the negative control pcAb or without pcAb (Fig 5A and 5B). However, positive indirect immunofluorescence was detected in the presence of rHspA-GGT pcAb (Fig 5C). These results indicate rHspA-GGT pcAb is able to bind *H*. *pylori* 26695 directly *in vitro*. As both HspA and GGT are involved in *H*. *pylori* colonization and growth in the gastric mucosa, antibody binding may impact the biological function of these proteins and may also be essential for bacterial clearance. The limitation of this experiment is that a positive control was not used to identify *H*. *pylori*, as no antibody against this bacterial was market available for the moment.

## Discussion

Vaccines efficacy relies largely on the antigens used for immunization. In this paper, the efficacy of two antigens was tested. HspA, a member of the GroES chaperone family, is a 13 kDa cytoplasmic protein that also located on the bacterial cell surface [20]. Compared to its homologue, HspA harbors a unique C-terminal domain consisting of 28 residues, which is thought to be crucial for binding metal ions [20]. Research suggests that HspA is involved in nickel sequestration, and serves as a specialized nickel donor protein for urease. In this way, HspA is thought to be essential for urease activity and bacterial colonization [21]. In addition, HspA is able to bind Bi^3+^, altering the protein’s quaternary structure, and converting from a heptamer to a dimer [22]. Thus, HspA may be a potential target for the bismuth anti-ulcer drug when used against *H*. *pylori*. Importantly, research also indicates HspA exhibits strong antigenic properties [14].

The second antigen, GGT, is constitutively expressed in all *H*. *pylori* strains and has gained attention recently as a formidable virulence factor [23]. Firstly, GGT is an important colonization factor strongly associated with the development of peptic ulcers [24]. Secondly, it is able to induce apoptosis and necrosis of gastric epithelial cells by induction of cell cycle arrest, production of reactive oxygen species, and secretion of inflammatory cytokines such as IL-8 [25]. Finally, GGT has immunomodulatory functions. GGT is involved in inhibition of T cell-mediated immunity and dendritic cell differentiation [26]. GGT is directly involved in the pathogenesis of *H*. *pylori*, and this feature makes it a promising vaccine component.

The importance of adjuvants in effective vaccine development has been discussed elsewhere [27]. In this study, LTB and CpG were selected as adjuvants to enhance the immune response against *H*. *pylori* infection. The former is a mucosal adjuvant [28], whereas as the latter induces a cell-mediated response [29]. Immunization with antigens supplemented with LTB elicited a balanced Th1/Th2 response and a strong local sIgA response, whereas antigen supplemented with CpG induced a Th1-biased immune response. Both adjuvants triggered the secretion of cytokines such as IFN-γ, IL-4, IL-5, and IL-17A by murine splenic cells, but CpG appeared to be more effective in inducing secretion of IFN-γ. Both adjuvants exhibited significant differences in bacterial clearance compared to immunizations performed without adjuvants (P<0.0001). Here, LTB was more effective than CpG (P<0.0001). These results highlight the importance of mucosal adjuvants in effective vaccine development against *H*. *pylori* infection.

What type of immunity is crucial for an effective *H*. *pylori* vaccine? The answer to this question, essential to vaccine design, remains controversial. In terms of the systemic humoral response, earlier studies have suggested that vaccines that induce an IgG antibody response may be involved in protection [30]. More recent research, however, has shown that although antibodies are detected at high titer following immunization, they are not required for protection [31]. In the present study, similar levels of serum IgG antibody were detected in mice immunized with rHspA-GGT supplemented with either LTB or CpG adjuvant. The two adjuvants, however, exhibited different capacities for mediating bacterial clearance (P<0.01). Thus, whether a high level of IgG antibody is required for vaccine protection remains unclear. We also evaluated the local humoral response by determining the level of gastric and intestinal sIgA. Significantly higher levels of sIgA were detected in mice immunized with rHspA-GGT supplemented with LTB, but not rHspA-GGT supplemented with CpG. Furthermore, sIgA levels were also correlated with reduced bacterial colonization, suggesting a local sIgA response may be essential for bacterial clearance. These results were consistent with earlier reports indicating higher mucosal IgA levels were associated with low bacterial density, suggesting a protective role for sIgA during *H*. *pylori* infection [32,33].

It has long been reported that cell-mediated immunity also confers protection against *H*. *pylori* infection in the absence of B cells [34]. However, the types of T cell responses that contribute to protective immunity remain unclear. Mohammadi M *et al* [35] reported that Th1 cells enhance the severity of gastritis and Th2 cells reduce bacterial load, but other research groups have emphasized the importance of the Th1 cell response in protective immunity [36,37]. There is also evidence that a Th17 response benefits bacterial growth [38], and that the CD8+ T cell response is also induced during *H*. *pylori* infection [39]. In our study, as indicated by the ratio of IgG1/IgG2a and measured cytokine concentrations, a complex Th1/Th2/Th17 response was observed in all immunized groups. This response was observed for all groups except the immunization with rHspA-GGT and CpG, which induced a Th1-biased immune response. It is difficult to evaluate which cell-mediated responses are predominant in vaccine protection. Further studies are required to fully evaluate the role of these cells in cell-depleted mice.

In a previous study, decreased expression levels of IL-10 and IL-4 were observed upon *H*. *suis* rGGT immunization, correlating with reduced gastric colonization [15]. In our study, however, levels of both IL-4 and IL-5 increased after rGGT immunization. These contradictory results may be caused by the use of different methods in these studies. In the former study, IL-10 and IL-4 levels were assessed by RT-qPCR using cDNA synthesized from stomach tissue. In the present study, IL-4 and IL-5 levels were determined by performing ELISA on the supernatants of antigen-stimulated spleen cells.

In summary, a recombinant subunit vaccine (rHspA-GGT) was designed and constructed in this study. Immunization with this protein and LTB adjuvant correlates with reduced bacterial colonization in the stomachs of mice, which may be mediated by a balanced Th1/Th2 CD4+ T cell response and a local humoral immune response. These results indicate that both HspA and GGT are promising candidates for development of subunit vaccines against *H*. *pylori* infection.

## Supporting Information

S1 Table Primers used in this study. (DOC)
