# Supplementary material for: Immunization with Heat Shock Protein A and γ-Glutamyl Transpeptidase Induces Reduction on the Helicobacter pylori Colonization in Mice
Source: PLoS One. 2015 Jun 23;10(6):e0130391. doi: 10.1371/journal.pone.0130391 (PMC4478016; doi:10.1371/journal.pone.0130391)
Supplement: S1 Table — (DOC) [file pone.0130391.s001.doc]

S1 Table. Primers used in this study.

| Constructs | Primer | Sequence of primers | Notation |
| --- | --- | --- | --- |
| rHspA | sense | 5’ GGAATTC*CATATG*AAGTTTCAGCCAT 3’ | *NdeI* |
|  | Anti-sense | 5’ CCG*CTCGAG*GTGTTTTTTGTGATCTG 3’ | *XhoI* |
| rGGT | sense | 5’ GGAATTC*CATATG*ACGCATTATTCTGT 3’ | *NdeI* |
|  | Anti-sense | 5’ CCG*CTCGAG*AAATTCTTTCCTTGGATC 3’ | *XhoI* |
| rHspA-GGT | P1 | 5’ GGAATTC*CATATG*ACGCATTATTCTGT 3’ | *NdeI* |
|  | P2 | 5’ AACTTCAT*CTTCTT*AAATTCTTTCCTTGG 3’ | *“KK” linker* |
|  | P3 | 5’ AAGAATTT*AAGAAG*ATGAAGTTTCAGCCA 3’ | *“KK” linker* |
|  | P4 | 5’ CCG*CTCGAG*GTGTTTTTTGTGATCTG 3’ | *XhoI* |
